# Financial implications of protocol-based hypertension treatment: an insight into medication costs in public and private health sectors in India

**DOI:** 10.1038/s41371-022-00766-x

**Published:** 2022-10-21

**Authors:** Swagata Kumar Sahoo, Anupam Khungar Pathni, Ashish Krishna, Bhawna Sharma, Danielle Cazabon, Andrew E. Moran, Dagmara Hering

**Affiliations:** 1 Resolve to Save Lives, New Delhi, India; 2 Resolve to Save Lives, New York, NY USA; 3 Columbia University Irving Medical Center, New York, NY USA; 4 Department of Hypertension and Diabetology, Medical University of Gdansk, Gdansk, Poland

**Keywords:** Hypertension, Disease prevention

## Abstract

Hypertension is a major public health challenge in low- and middle-income countries (LMICs) and calls for large-scale effective hypertension control programs. Adoption of drug and dose-specific treatment protocols recommended by the World Health Organization-HEARTS Initiative is key for hypertension control programs in LMICs. We estimated the annual medication cost per patient using three such protocols (protocol-1 and protocol-2 with Amlodipine, Telmisartan, using add-on doses and different drug orders, adding Chlorthalidone; protocol-3 with a single-pill combination (SPC) of Amlodipine/Telmisartan with dose up-titration, and addition of Chlorthalidone, if required) in India. The medication cost was simulated with different hypertension control assumptions for each protocol and calculated based on prices in the public and private sectors in India. The estimated annual medication cost per patient for protocol-1 and protocol-2 was $33.88–58.44 and $51.57–68.83 for protocol-3 in the private sector. The medication cost was lower in the generic stores ($5.78–9.57 for protocol-1 and protocol-2, and $7.35–9.89 for protocol-3). The medication cost for patients was the lowest ($2.05–3.89 for protocol-1 and protocol-2, and $2.94–3.98 for protocol-3) in the public sector. At less than $4 per patient per annum, scaling up a hypertension control program with specific treatment protocols is a potentially cost-effective public health intervention. Expanding low-cost generic retail networks would extend affordability in the private sector. The cost of treatment with SPC is comparable with non-SPC protocols and can be adopted in a public health program considering the advantage of simplified logistics, reduced pill burden, improved treatment adherence, and blood pressure control.

## Introduction

In India more than 200 million adults have hypertension, but less than one-tenth of the hypertensive population have their blood pressure (BP) controlled [1, 2]. Uncontrolled BP remains a major risk factor for cardiovascular disease (CVD), contributing to an estimated 1.6 million deaths annually in India, mostly due to ischemic heart disease and stroke [3]. The world economic forum estimated over $2 trillion in losses to the Indian economy due to CVD between 2012 and 2030 [4]. This requires the rapid upscaling of public health programs to reduce the burden of hypertension and associated CVD risk. Adoption of drug and dose-specific hypertension treatment protocols is considered key to scaling up programs in resource-limited settings including India [5]. By providing a step‐by‐step schedule for initiation, titration, and addition of medications, this approach facilitates the simplicity, scale, and speed of program implementation. Treatment protocols are useful in decentralizing hypertension management in primary healthcare to improve treatment coverage and BP control by reducing clinical variability and therapeutic inertia. The India Hypertension Control Initiative0 (IHCI) has adopted standardized drug- and dose-specific hypertension treatment protocols recommended in the World Health Organization (WHO)-HEARTS package [6, 7]. However, the implementation and sustainability of this strategy would require a financial assessment to inform public funding and policy decisions that can improve care for the hypertensive population and access to affordable BP lowering medicines. Economic analysis can guide priority setting for hypertension management [8–10]. Hypertension treatment can be cost-effective by implementing simple population management programs for primary prevention. Expanding national insurance coverage, particularly for the primary prevention of CVD is expected to have a substantial impact and reasonable cost-effectiveness in India [10]. In this study, therefore, we estimated the annual medication cost of protocol-based hypertension treatment per patient, and the comparative cost of alternative treatment protocols in the public and private sectors in India.

## Method

### Selection of hypertension treatment protocol

To estimate the annual medication cost for the treatment of hypertension, we used treatment protocols aligned with those recommended in the WHO-HEARTS technical package [6]. Three alternative drug and dose-specific treatment protocols consisting of a calcium channel blocker (CCB), an angiotensin receptor blocker (ARB), and a diuretic were selected for this study (Table 1). Protocols 1 and 2 adopted a five-step escalation approach and the recommended use of Amlodipine (CCB), Telmisartan (ARB), and Chlorthalidone (diuretic). Under protocols 1 and 2, treatment is initiated with Amlodipine 5 mg followed by two different add-on pathways. For patients with uncontrolled hypertension (defined as systolic BP ≥140 mmHg or diastolic BP ≥90 mmHg), protocol-1 recommends escalation to Amlodipine 10 mg at step 2, prescription of Telmisartan 40 mg at step 3, then escalated to Telmisartan 80 mg at step 4 and finally the addition of Chlorthalidone 12.5 mg at step 5. Protocol-2 recommends the addition of Telmisartan 40 mg at step 2, escalation to Telmisartan 80 mg at step 3 before escalating to Amlodipine 10 mg at step 4, and addition of Chlorthalidone 12.5 mg at step 5. Protocol-1 and Protoco1-2 have already been adopted by several Indian states under the national hypertension control program. Protocol-3 is based on the “adapted example of Telmisartan 40 mg/Amlodipine 5 mg as a single-pill combination (SPC) regimen” recommended by the WHO-HEARTS technical package, which is a four-step protocol using an SPC of Amlodipine 5 mg with Telmisartan 40 mg. Treatment is initiated with a half SPC tablet, followed by an increase to one SPC tablet, and then two SPC tablets at step 3, and subsequent addition of Chlorthalidone 12.5 mg at step 4 if BP remains uncontrolled.

**Table 1:** Drugs included in the hypertension treatment protocols.

Steps	Protocol-1 (AATTC)	Protocol-2 (ATTAC)	Protocol-3 with single pill combination (SPC) of A + T
1	Initiate treatment with Amlodipine 5 mg	Initiate treatment with Amlodipine 5 mg	Initiate treatment with 1/2 (half) pill SPC Amlodipine 5 mg with Telmisartan 40 mg
2	Escalate to Amlodipine 10 mg	Add Telmisartan 40 mg	1 Pill of SPC Amlodipine 5 mg with Telmisartan 40 mg
3	Add Telmisartan 40 mg	Escalate to Telmisartan 80 mg	2 Pills of SPC Amlodipine 5 mg with Telmisartan 40 mg
4	Escalate to Telmisartan 80 mg	Escalate to Amlodipine 10 mg	Add Chlorthalidone 12.5 mg
5	Add Chlorthalidone 12.5 mg	Add Chlorthalidone 12.5 mg	NA

Source: HEARTS technical package for cardiovascular disease management in primary healthcare: evidence-based treatment protocols. Geneva: WHO; 2018 (WHO/NMH/NVI/18.2). License: CC BY-NC-SA 3.0 IGO.

Escalation to a higher step of treatment protocol is recommended when blood pressure remains uncontrolled (≥140 mmHg or ≥90 mmHg) with the existing treatment protocol step.

AATTC/ATTAC are mentioned as a short form for protocols 1 and 2. The alphabet reflects the step-up sequence.

### BP control assumptions for each treatment protocol

We adopted multiple scenarios for treatment stepwise hypertension control assumption based on initial findings from hypertension treatment and BP control in a cohort of 24 sentinel site clinics in India under the IHCI [7]. For each hypertension treatment protocol, four treatment stepwise scenarios for achieving BP control were used ranging from conservative to more optimistic assumptions. The adopted scenarios are detailed in Table 2. Protocol-1 and protocol-2 followed a five-step approach, whereas the protocol with SPC (protocol-3) has only 4 steps. The treatment stepwise BP control rate for SPC (protocol-3) was kept aligned with the single-molecule protocol (protocol-1 and protocol-2) based on the drug and dose regimen prescribed.

**Table 2:** Scenarios of step-wise hypertension control assumption under selected treatment protocol.

Protocol	Scenario 1	Scenario 2	Scenario 3	Scenario 4
Protocol 1 (AATTC)	30:30:20:10:10	40:25:15:10:10	50:20:15:10:5	60:15:13:7:5
Protocol 2 (ATTAC)	30:30:20:10:10	40:25:15:10:10	50:20:15:10:5	60:15:13:7:5
Protocol 3 (with SPC)	30:30:30:10	40:25:25:10	50:20:25:5	60:15:20:5

The scenarios described the expected proportion of patients having their blood pressure controlled at each step of the treatment or required to be treated as per the medication regimen of the protocol step. E.g. scenario 1, 30:30:20:10:10 suggests, 30% of patients will require to be treated as per step 1 medication regimen, another 30% of patients will require to be treated according to step 2, and only 10% of patients would require the escalation to 5th step of the treatment protocol. Patients with uncontrolled blood pressure with the use of the 5th step may need additional medication, but all these patients will need drug therapy prescribed at step 5. All described scenarios consider 100% patients.

### Calculation of antihypertensive drug requirement and the cost of treatment per patient per year

The requirement of each protocol drug per patient per year was calculated for each of the simulated scenarios for all three hypertension treatment protocols using the MS-Excel-based tool. The monthly (30-day) drug requirement (in a number of pills) per 100 patients was calculated considering the proportion of patients to be treated at different steps of the treatment protocol for all 4 scenarios for each protocol and the annual requirement per patient was extrapolated accordingly. Table 3a and b demonstrate the drug requirement calculation for protocol-1 and scenario-1.

**Table 3:** (a) Calculation of average antihypertensive drug requirement per patient per month in a number of pills, protocol 1 (AATTC), scenario 1. (b) Estimated antihypertensive drug requirement per patient in a number of pills.

Monthly antihypertensive drug requirement matrix of 100 patients								
Treatment Stage	Expected % control	A5	2*A5	Total A5 requirement	T40	2*T40	Total T40 requirement	CTD 12.5
**(a)**								
1	30	900		900			0	
2	30		900	1800			0	
3	20		600	1200	600		600	
4	10		300	600		300	600	
5	10		300	600		300	600	300
Total	100	900	2100	5100	600	600	1800	300

(b)							
	**A5**	2*A5	**Total A5 requirement**	**T40**	2*T40	**Total T40 requirement**	**CTD 12.5**
Monthly requirement	9	21	51	6	6	18	3
Annual requirement			621			219	37

*A5* amlodipine 5 mg tablet, *T40* telmisartan 40 mg tablet, *CTD 12.5* chlorthalidone 12.5 mg tablet.

In India, the medication cost varies between the public and private sectors. Prices for generics are also lower compared to brand-name antihypertensive medicines in the private sector. Thus, we considered all the above settings to evaluate the annual cost of medication. The cost of selected drugs in each protocol was obtained from the private (most prescribed brands and generic drugs) and public sectors. The public procurement prices were collected from the Ministry of Health and Family Welfare, Government of India’s Drug and Vaccine Delivery Management System central dashboard [11]. Prices for each protocol drug for most prescribed brands in India’s private healthcare sector were collected from our previous study on the price of antihypertensive drugs and the prices of low-cost generic drugs were sourced from the Jan Aushadhi website [12, 13].

With the annual drug requirements for all protocols and scenarios and the known drug cost (price per pill), medication cost per patient per annum was calculated by “multiplying the annual requirement of each drug by their cost and summing them together. The following formula was used:

cost per patient per annum = (an annual requirement for Amlodipine in a number of pills *cost per pill of Amlodipine + annual requirement for Telmisartan in number of pills *cost per pill of Telmisartan + annual requirement for Chlorthalidone in number of pills *cost per pill of Chlorthalidone).

The estimated annual medication cost for hypertension treatment was calculated for all three protocols for each treatment scenario for (i) most prescribed brand drugs in the private sector, (ii) generic medicines in the private sector– Jan Aushadhi, and (iii) drugs used in the public health programs.

## Results

### Cost of selected hypertension protocol drugs

The median price per pill of the best-selling Amlodipine 5 mg tablets in the private sector is INR 2.8 (3.78 cents), whereas the cost of the generic brand in the Jan-Aushadhi store is INR 0.5 (0.68 cents), and the prevalent large scale public procurement price is INR 0.14 (0.19 cents). Similarly, for Telmisartan 40 mg the median prices of the best-selling brand in the private sector, at Jan-Aushadhi stores and the prevalent public procurement prices are INR 7.19 (9.72 cents), 1.1 (1.49 cents), and 0.5 (0.68 cents) per pill, respectively. The cost of the selected protocol drugs for most prescribed brands in the private market, generic retail—Jan- Aushadhi store, and the public sector are summarized in Table 4. In India, there are multiple levels of stocking, and reselling with a structure of profit margin in the private sector, whereas procurement in the public sector is placed directly from manufacturers following competitive tenders, leading to the public procurement of medicines at a much lower price. In our study, all costs presented are the ultimate cost per patient. In the public sector medicines are provided by the government and patients receive them for free. Hence, the medication cost mentioned for the public sector is equivalent to the cost of which government buys the drugs.

**Table 4:** Cost of selected drugs (price per pill in INR) in the private, generic retail, and public sectors.

Drug class	Molecule	Strength	No. of brands considered^a^	MRP range per tablet (INR) Private sector	Median price per tablet (INR) Private sector	Jan Aushadhi price (Generic retail)	Median Public proc. Price
ARB	Telmisartan	40 mg	12	3.40–7.33	7.19	1.1	0.5
CCB	Amlodipine	5 mg	10	0.68–2.86	2.8	0.5	0.14
Diuretic	Chlorthalidone	12.5 mg	7	2.00–6.86	5.79	1.3	0.7
CCB + ARB	Amlodipine + Telmisartan	5 mg/40 mg	13	3.35–14.30	10.7	1.5	0.59

Prices for the private sector market are in 2019–2020 India rupees (INR). Recent prices of low-cost generic drug options (Jan Aushadhi), accessed in July 2021.

*ARB* angiotensin receptor blocker, *CCB* calcium channel blocker.

^a^Brands that account for more than 70% of the total market share by volume in the private sector, were considered to get a price range and a median price in the private sector.

### Average annual requirement of hypertension protocol drugs per patient

For the selected protocols 1 and 2, the total number of pills required per patient per annum (including Amlodipine 5 mg, Telmisartan 40 mg, and Chlorthalidone 12.5 mg) ranged from 664 pills to 877 pills depending on the proportion of patients having their BP controlled at each step of the treatment protocol. The total pill requirement can be lowered and range between 530 and 657 pills per patient per annum if, a double dose of Amlodipine is in one tablet (i.e. Amlodipine 10 mg instead of two tablets of Amlodipine 5 mg) and a double dose of Telmisartan (i.e. Telmisartan 80 mg instead of two tablets of Telmisartan 40 mg) is prescribed for patients who need the maximum drug dose. However, the use of multi-dose tablets can add complexity to public health supply chains as more items are required to be ordered and processed.

When adopting the protocol with SPC of Amlodipine 5 mg with Telmisartan 40 mg (Protocol 3), the annual requirement of pills per patient is significantly reduced, ranging between 365 and 493 pills only, suggesting that the majority of patients may achieve their BP control with just one tablet a day. The approximate annual requirement of each drug for all four scenarios for all three selected protocols is illustrated in Table 5.

**Table 5:** Estimated annual requirement of drugs per patient and annual cost of medication (in USD) for hypertension treatment with the use of selected protocols.

Protocol	Scenario	Treatment stage-wise control assumption	Approximate annual drug requirement per patient				Annual cost of medication per patient in USD		
			Amlodipine 5 mg	Telmisartan 40 mg	Chlorthalidone 12.5 mg	Total pill requirement	Private sector	Private low-cost generic	Govt. program
Protocol 1: AATTC	1	30:30:20:10:10	621	219	37	876	47.61	8.09	3.00
	2	40:25:15:10:10	584	201	37	821	44.46	7.57	2.81
	3	50:20:15:10:5	548	164	18	730	38.10	6.46	2.32
	4	60:15:13:7:5	511	135	18	664	33.88	5.78	2.05
Protocol 2: ATTAC	1	30:30:20:10:10	438	402	37	876	58.44	9.57	3.89
	2	40:25:15:10:10	438	347	37	821	53.12	8.76	3.52
	3	50:20:15:10:5	420	292	18	730	45.68	7.50	2.94
	4	60:15:13:7:5	409	237	18	664	39.95	6.61	2.55
	**Scenario**	**Treatment stage-wise control assumption**	**Amlodipine 5** **mg** + **Telmisartan 40** **mg SPC**		**Chlorthalidone 12.5** **mg**	**Total pill requirement**	**Private sector**	**Private low-cost generic**	**Govt. program**
WHO-HEARTS SPC protocol 3	1	30:30:30:10	456		37	493	68.83	9.89	3.98
	2	40:30:20:10	420		37	456	63.55	9.15	3.69
	3	50:20:25:5	383		18	402	56.84	8.09	3.23
	4	60:15:20:5	347		18	365	51.57	7.35	2.94

Exchange rate USD: INR used is 1:74.

### Annual cost of medication per patient for hypertension treatment

The approximate annual cost of antihypertensive medication per patient in the private sector ranges between $33.88 and 58.44 when using protocol-1 and protocol-2, and between $51.57–68.83 when using SPC (protocol-3). When drugs are purchased at low-cost generic stores (Jan-Aushadhi stores), the cost of medication per patient per annum was substantially reduced, ranging between $5.78 and 9.57 for protocols 1 and 2, and $7.35–9.89 for SPC (protocol-3). The annual medication cost per patient in the public sector, where drugs are procured by the state on a large scale through competitive tenders is substantially lower, ranging between $2.05 and 3.89 for protocol-1 and protocol-2 and $2.94–3.98 for protocol-3 (Table 5).

## Discussion

Our study is the first that estimated the annual medication cost for three different treatment protocols adopted from the WHO-HEARTS for controlling BP in a large proportion of patients with uncomplicated hypertension in India. Our study showed that the medication cost following a hypertension treatment protocol-based approach can be as low as $2–4 per patient per annum at the program level in the public sector in India. While drugs are provided to the patient free of charge in the public sector, the cost to the state is less than 1/15th of the cost that a patient has to pay for purchasing them in the private sector.

Our results on medication cost are striking and much lower than previous studies on estimating the cost of hypertension treatment or economic modeling studies on hypertension management. Previous modeling exercise studies have shown that implementing hypertension control strategies in low-resource settings depends on cost consideration but is a cost-effective intervention for low- and middle-income countries (LMICs) and can be cost-effective in the public sector in India, mostly due to low medication costs [14, 15]. The WHO Global Strategy for the prevention and control of NCDs (cardiovascular disease, cancer, diabetes, and chronic respiratory disease) reported that the estimated annual per capita cost for scaling up population-based health interventions addressing NCD risk factors (tobacco use, alcohol use, physical inactivity, unhealthy diet) is under $1 in low-income countries and $1.5 in LMICs over a period between 2011 and 2025 [8]. However, the annual medication cost of a multidrug hypertension regimen per treated person with hypertension at high risk for CVD was estimated to be higher accounting for $70 in low-income countries and $84 in LMICs. Notably, the WHO estimated cost has been based on past data reflecting international supplier medicines prices which are lower than retail prices and represent the cost to the government, not the patient. The medication cost can vary depending on location, time, and analytic method used. Furthermore, gaps in evidence exist on program elements that can affect cost-effectiveness in LMICs, such as standardized treatment protocols [14]. A recent study expanding the WHO HEARTS costing tool application found that the total annual cost for the hypertension control program is estimated at $2.8 per capita in Bangladesh which can be used for further cost-effectiveness analysis to support evidence-based decision making for CVD prevention programs [16].

Further support for adequate pharmaceutical management comes from a comparison study across 36 countries using WHO/Health Action International data, emphasizing the need of focusing attention and financing on making cardiovascular medicines, particularly antihypertensive drugs, accessible in the public sector by promoting the use of generic medicines and reliable sourcing, and methods financing to prevent excessive margins in the supply chain [17].

The cumulative economic costs of premature death and disability from CVD in the LMICs are estimated to be US $3.7 trillion between 2011 and 2025 years, which is an average of 2% of LMIC GDP [18]. With limited data available on the cost of drugs for hypertension treatment in India, our study shows that scaling up a protocol-based hypertension control program in the public sector can be a highly cost-effective intervention with significantly lower expenditure on hypertension treatment, a major contributor to CVD morbidity and mortality.

Given that most hypertensive patients require at least two or more drugs for their BP control, fixed-dose combinations, also referred to as SPCs are increasingly recommended for the initial hypertension treatment [19–21]. In 2019 the WHO included four SPC antihypertensive drugs on its essential medicines list (EML). However, despite their recommendation in the national hypertension treatment guidelines [22] and abundant availability in the Indian market [23], there is limited availability of antihypertensive SPC drugs in the public health sector as India has not yet included antihypertensive SPC medications in the National EML [24].

Therapy with SPCs reduces pill burden, increases patient adherence and/or persistence, and helps in rapid improvement in BP control when compared to the same dose of drugs given alone [21]. Our analysis showed that the cost of drugs in the public sector following the adoption of SPC (protocol 3) largely overlaps with the cost of medication with non-SPC protocols. Therefore, antihypertensive SPC medicines as recommended by the WHO should be included in the National EML in India and the public sector should consider the adoption or transition to an SPC-based protocol for hypertension control programs.

Our study found that the cost of medication is ~25% higher with the SPC protocol in the private sector. The inclusion of SPCs under the Drug Price Control Order, particularly for those SPCs whose individual components are already under price control, is likely to improve their affordability in the private sector [25]. While controlling the price of drugs has the potential to increase consumer welfare by setting a price cap, establishing a price ceiling can also involve trade-offs. Too low a price cap may discourage pharmaceutical companies from joining or continuing to operate in the market. Price control is a double-edged sword that should be used with caution with two key objectives – industrial development and consumer interest.

When drugs are prescribed in generic name and patients have the access to purchase them from a generic pharmacy such as the Jan-Aushadhi stores, our study showed that the cost of hypertension protocol drugs per patient per annum drops substantially to just $6–10, which is up to 80% lower compared to drugs prescribed under the brand names and needs to be purchased at the private retail pharmacy. Although Jan-Aushadhi stores are not widely available which is a significant limitation of the healthcare system in India, the network is rapidly growing. With the potential for such a massive reduction in out-of-pocket expenses on drugs through access to such generic drug stores (Jan-Aushadhi stores), increased public presence and awareness of these stores can significantly improve the affordability of drugs in the private sector.

Promoting the treatment of hypertension using drug-dose-specific treatment protocols as recommended by the WHO-HEARTS technical package is effective and simple to implement in primary care and has practical advantages over existing comprehensive treatment guidelines. Numerous LMICs including India, Bangladesh, Ethiopia, Nigeria, and the Philippines have adopted such protocols for hypertension management in general practice and many would be planning in the same line. India has the advantage of low medication costs due to its large manufacturing base and huge domestic market. The conditions in other LMICs may not be the same, and the availability of drugs and the cost-effectiveness of hypertension treatment would differ from country to country. However, the approach outlined in this study can be used in budget planning to scale up national and regional hypertension control programs. By considering the population and hypertension prevalence, program managers can estimate the total hypertensive population. Since not all patients would be enrolled in a public sector hypertension control program, the state and/or the country may establish a target and timeline for enrollment based on the health-seeking behavior and public health infrastructure to progressively expand treatment coverage. Alternatively, countries with limited financial resources may plan to scale up the program gradually, based on fund availability.

While pooled procurement of generic drugs as a part of a competitive procurement process is recommended to reduce costs, it is important that purchasing agencies, and governments implementing public health programs have quality assurance systems in place to purchase drugs from a reliable source, verify drug quality while receiving and maintaining high-quality drugs throughout the storage and distribution process until they are received by patients.

## Limitations

Our study estimated the annual medication cost for hypertension treatment in India considering only three treatment protocols which could be a potential limitation. However, the proposed protocols follow the recommendation of the WHO-HEARTS package and hypertension guidelines [6, 21, 26] and are intended to meet the needs of a wide range of patients with uncomplicated arterial hypertension. Secondly, while the protocol adopted elsewhere and the cost range of drugs may be different, the same methodology used in the present study can help to estimate the medication cost in other LMICs. Thirdly, some proportion of patients may require hypertension treatment beyond the selected protocol and the cost of advanced treatment protocols is not included in the present analysis. Fourthly, our study estimates the medication cost only and does not include the cost of the periodic investigation. Finally, protocol-1 and protocol-2 use single molecule drugs which may affect medication adherence. However, the initiation of hypertension therapy with a single molecule drug is also recommended and can help to determine the potential side effects of a specific drug class. Medication adherence and clinical inertia can be improved with the implementation of the proposed protocol-3 with SPC therapy leading to better BP control and outcomes. Our study simulated a wide range of assumptions from best case to more conservative case scenarios to obtain a range of drug costs per patient per year.

## Conclusion

To achieve hypertension control targets, countries will need to increase public sector coverage and treatment affordability in the private sector. While standardized drug-dose-specific treatment protocols are key to scaling up hypertension control programs by allowing the decentralization of treatment to the primary health care level, they are also a cost-effective approach in terms of better affordability and preventing the higher risk of CVD-related complications. This study provides an overview of the method for estimating budget requirements for medications that can assist in informed protocol selection and allocate prudent resources to strengthen hypertension control programs in the public sector in both urban and rural areas in India. The findings and recommendations of this study are directly relevant to the public policy context aimed at reducing the burden of CVD, in particular hypertension, premature deaths from CVD, and the even more ambitious targets under the Sustainable Development Goals.

### Summary table

#### What is known about topic

- Hypertension is the leading cause of premature death worldwide, with the highest age-standardized death from CVD and hypertension reported in LMICs.
- Large-scale hypertension control programs with public health approach are essential to reducing the hypertension burden.
- Adopting drug-dose-specific treatment protocols is a key strategy for improving BP control in resource-limited settings.

#### What this study adds

- A practical approach to estimate the cost of medication per patient per annum for protocol-based hypertension care.
- An insight into changes in drug costs based on the treatment protocol adopted and sources of purchase of antihypertensive drugs.
- A perspective on public funding plans for large-scale hypertension control programs and policy decisions that can improve medication affordability.

## Data Availability

The datasets used and/or analyzed that support the findings of this study are available from the corresponding author on reasonable request.
